# Real-time personalized feedback in mHealth for adolescents

**DOI:** 10.1177/20552076241247937

**Published:** 2024-05-15

**Authors:** Evelien Dietvorst, Manon HJ Hillegers, Jeroen S Legerstee, Lianne P De Vries, Annabel Vreeker, Loes Keijsers

**Affiliations:** 1 Department of Child and Adolescents Psychiatry/Psychology Erasmus MC Sophia Children's Hospital, Erasmus University Medical Center, Rotterdam, The Netherlands; 2 Erasmus School of Social and Behavioural Sciences Department of Psychology, Education & Child Studies Erasmus University, Rotterdam, The Netherlands

**Keywords:** Feedback, self-monitoring, experience sampling method, eHealth, ecological momentary intervention

## Abstract

Mobile Health (mHealth) interventions have the potential to improve early identification, prevention, and treatment of mental health problems. Grow It! is a multiplayer smartphone app designed for youth aged 12–25, allowing them to monitor their emotions and engage in daily challenges based on Cognitive Behavioral Therapy (CBT) principles. Recently, a personalized mood profile was added to improve the app. We investigated whether real-time personalized feedback on mood enhances app engagement, user experience, and the effects on affective and cognitive well-being.

Sample A (*N* = 1269, age = 18.60 SD = 3.39, 80.6% girls, 95.4% Dutch) played the original app without feedback on their mood, and an independent Sample B (*N* = 386, age = 16.04 SD = 3.21, 67.6% girls, 82.9% Dutch) received the renewed version with personalized real-time feedback on their mood.

Participants who received personal feedback did not have higher app engagement (*t*(1750,400) = 1.39, *P *= .206, *d* = 0.07; *t*(692,905) = 0.36, *P *= .971, *d* = 0.0) nor higher user experience (*t*(177,596) = 0.21, *P *= .831, *d* = 0.02; (*t*(794) = 1.28, *P *= .202, *d* = 0.12; χ^2^ (659,141) = 2.83, *P *= .091). Players of the renewed version (Sample B) experienced significant improvements in affective (*t*(175) = 3.01, *P = *.003, *d* = 0.23) and cognitive well-being (*t*(175) = 3.48, *P *= <.001, *d* = 0.26) over the course of three weeks.

The renewed version Grow It! has the potential to enhance youths’ affective and cognitive well-being. However, adding real-time insights did not seem to affect app engagement nor user experience.

## Introduction

Ever since the COVID-19 pandemic there has been a steady increase in children anxiety and depression symptoms across the globe. The need for child and adolescent mental health services increasingly outweighs the resources such as the availability of trained health-care professionals resulting in long waiting lists.^1^ Clearly, innovations are needed to prevent a further rise of mental ill health among the youth. Innovative mobile health applications (mHealth) have the potential to prevent the emergence of problems, and improve early identification and treatment of mental health problems.^2, 3^ mHealth comes with important benefits compared to the traditional therapy. First, the accessibility of mHealth applications is not limited by professional resources. Second, with almost all adolescents having access to their own mobile device, mHealth is scalable and accessible, and adolescents can use it whenever and wherever they want. Thirdly, from a user perspective, adolescents may perceive mHealth as less stigmatizing compared to face-to-face health-care,^4^ and it may also have a better fit with their natural ways of interacting with others, which increasingly takes place online.^5, 6^ As such, mHealth may be a good alternative when traditional interventions are not possible. Even though the developments of mHealth interventions are rapidly evolving, still few evidence-based interventions are currently being used in practice. Earlier work on mHealth for adolescents has shown that it is feasible in youth^7^ and part of regular treatment for psychiatric symptoms such as depressive symptoms^8^ and psychotic symptoms.^7, 9^

However studies validating the effects of standalone mHealth on mental health and well-being are scarce.^8, 10^ Whereas a wide variety of mHealth tools are now successfully implemented in different clinical settings e.g.^11–13^ one of the major challenges of mHealth interventions is to keep adolescents motivated and engaged,^14^ especially when there is no professional involved. Earlier work has hinted at several factors which may improve this. First, co-creation of mHealth applications with youth has been suggested as a promising approach to improve the look, feel, and attractiveness of applications.^15, 16^ Second, providing real-time interactions with participants may be key. For instance, Bakker et al. (2016) recommend to optimize the effectiveness of mHealth for youth with internalizing problems by combining both self-monitoring of emotional well-being throughout the day^16^ (e.g. using Experience Sampling Method^17^) with real-time personalized feedback to improve motivation and engagement. As such, monitoring becomes an ecological momentary intervention (EMI)—insights and challenges are delivered in everyday life rather than only in a therapeutic session with a professional.^18^ Personalized feedback on participants’ own emotional well-being and real-time insight in the effects of their behavior might be both motivating and improve engagement and user experience which is also highlighted by Knaup et al. (2009)—this is the main hypothesis tested in this study.^19^ This hypothesis is tested by comparing two versions of the Grow It! app, one with and one without a personalized real-time mood profile.

### Real-time personalized feedback in the Grow It! app

**Figure 1.**
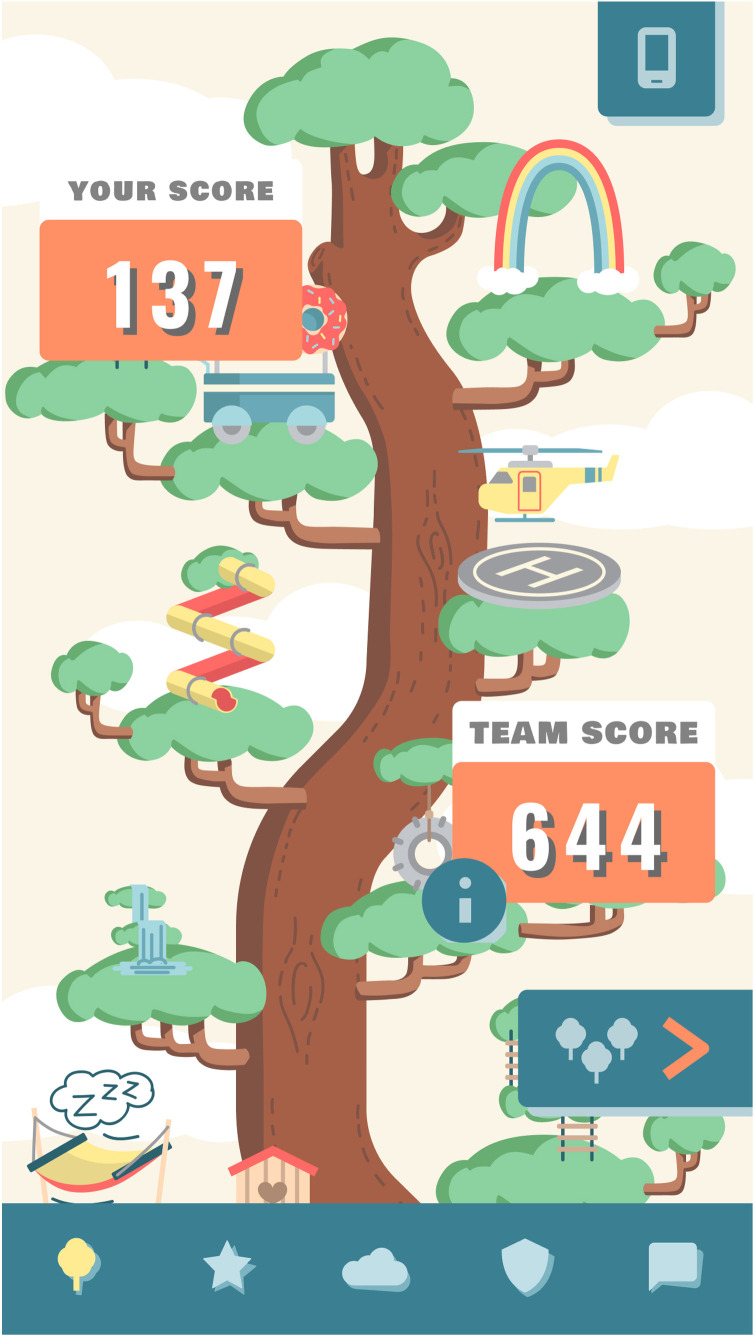
Grow It! app.

In short, the application central in this study is the multiplayer smartphone serious game Grow It! app [^10, 20^; Figure 1]. It is an ecological momentary intervention^18^ in which 12–25 year olds engage in two activities. (1) For 3 weeks, participants self-monitored their well-being with an Experience Sampling Method (ESM) component, and as such they gathered assessments about the emotional experiences, whereabouts, and companionships at five random moments per day. (2) Participants conducted daily challenges or assignments based on the enhancement of different coping strategies: distraction, problem solving, social support, and acceptance. An example of a challenge is as follows: ask someone what they like about you and write it down (assignment; aimed at social support). The development, feasibility, and acceptance of Grow It! was published elsewhere.^10^ The first version of the app was launched and tested on a large scale during the COVID-19 pandemic. Results revealed that in 1195 adolescents who had played this gamified mHealth tool, affective and cognitive well-being significantly increased, and depressive symptoms and anxiety decreased after 3–6 weeks.^20^

Based on the feedback from players, extensive user evaluations, literature review [e.g.^16^] as well as ongoing conversations with professionals, a third feature was added to Grow It! recently. (3) In the new version of Grow It! adolescents received a real-time personalized mood profile based on self-reported mood in the ESM.

### The current study

In this study, we compare the results of Grow It! in a version without a personalized mood profile to a version with a personalized mood profile. Methods and hypotheses were preregistered. We aim to answer the following research questions: RQ1) Does user engagement improve by adding personalized real-time feedback? We expect that users of the renewed version of Grow It! would (*H1a*) report higher experience sampling compliance (i.e. more questions answered) and (*H1b*) complete more daily challenges compared to users who did not receive feedback.

RQ2) Does user evaluation improve by adding personalized real-time feedback? We expected that (*H2a*) user evaluation, (*H2b*), evaluation of design, (*H2c*), and recommendability of the app to friends would be higher among users who received personalized feedback.

Furthermore, we aimed to replicate earlier findings^20^ about the potential effectiveness of Grow It! app (i.e an increase in affective and cognitive well-being pre–post measurement) in the sample with personalized feedback by answering:

RQ3) How did adolescents’ affective and cognitive well-being change after playing Grow It? We expected adolescents (*H3a*) to improve in their affective or cognitive well-being after playing the renewed version of Grow It!

Without a priori hypotheses (*H4a*), we asked: RQ4) How many adolescents have improved in their affective or cognitive well-being after playing the renewed version of Grow It! and RQ5) we inspected whether app engagement and user experience of the renewed Grow It! was related to changes in affective or cognitive well-being. We expected that (*H5a*) more app engagement and (*H5b*) higher user experience are related to stronger increases in affective and cognitive well-being. The current study had the overarching objective to evaluate whether real-time feedback about participants’ own emotional well-being could increase app engagement and user experience among adolescent mHealth users. Moreover, we also inspect whether we can replicate our earlier findings with regard to improvement of well-being after playing Grow It!

Finally, as an exploratory analysis to gain understanding of clinical future applications of Grow It!, we conducted nine semi-structured interviews with health-care professionals and explored their views regarding the added value of personalized feedback in an mHealth tool and to asses potential barriers seen by professionals for implementation in practice.

## Methods

### Participants, procedure and recruitment

Grow It! was developed according to cutting-edge guidelines for app development (see ^7^ for a detailed description). As such, with each study, we implement lessons from our own results and also the rapid developments in our field to further improve our design and effectiveness of the app. New users, then, play the improved version. In this study, we compare a version with a personalized mood profile to a version without a personalized mood profile. In each new phase of the developmental process, we apply the same design to allow comparison between different iterative improvement cycles: Grow It! is tested with an experimental study without a control group (pre–post design).

To test our hypotheses, we compare quantitative data from two independent community-based samples that come from a larger project (Grow It!). Sample A (*N* = 1269, age = 18.60 SD = 3.39, 80.6% girls, 95.4% Dutch, educational level low 17.9%, medium 35.1%, 46.6% high, 0.9% other) played the original version of the Grow It! app for 3 weeks without the personalized mood profile and could enroll in the app from December 2020 until February 2021 [see ^20^ for meta-data which were used here]. Sample B played the renewed version of the Grow It! app with personalized real-time mood profile for 3 weeks (*N* = 386, age = 16.04 SD = 3.21, 67.6% girls, 82.9% Dutch, educational level low 8.1%, 19.9% medium, 70.2% high, 1.8% other) and could enroll in the app from March 2021 until May 2021. Other than the addition of a mood profile, no changes were made. Data collection took place during the COVID-19 pandemic. During the enrolment periods, there were varying government measures in response to COVID-19, which fluctuated from strict lockdowns and curfews to loosened restrictions. Grow It! was initially designed for at-risk youth, such as those with a parent with a psychiatric disorder or a chronic somatic disease. However Grow It! became publicly available during the COVID-19 pandemic, considering all Dutch youth as “at risk for emotional problems.”

In each sample, similar recruitment strategies were conducted. To participate, adolescents (12–25 year olds) needed to be able to read and write Dutch, live in the Netherlands, and own a smartphone. Participants were recruited via advertisements and a promotion video on (social) media, through online announcements by schoolteachers [see ^20^, for details] and via teachers who used our educational materials. All study information was published on our website (www.growitapp.nl/corona), where it was also possible to contact the team of researchers and where participants (and their parents for 12–16 year olds) signed the informed consent on a secure webpage. As a reward system, a random group of participants, with at least 70% compliance, was drawn monthly to win a gift voucher worth €75, €50, €30, or €15 or Apple Airpods. The study was carried out completely online, without video calls or face-to-face contact to motivate participants.

The pre–post research design (and instruments) were identical in each sample.

Following a brief online baseline questionnaire covering demographic characteristics, depressive and anxiety symptoms, as well as affective and cognitive well-being, adolescents received a personal login code for the Grow It! app, which they engaged with for 3 weeks. Daily mood and whereabouts were assessed within the app. After this period, participants completed a follow-up questionnaire online, covering mental health and well-being outcomes as measured at baseline, along with detailed questions on their user experience with the Grow It! app. For a complete overview of all measures consult our online codebook.

### New feature: Real-time personalized mood profile

**Figure 2.**
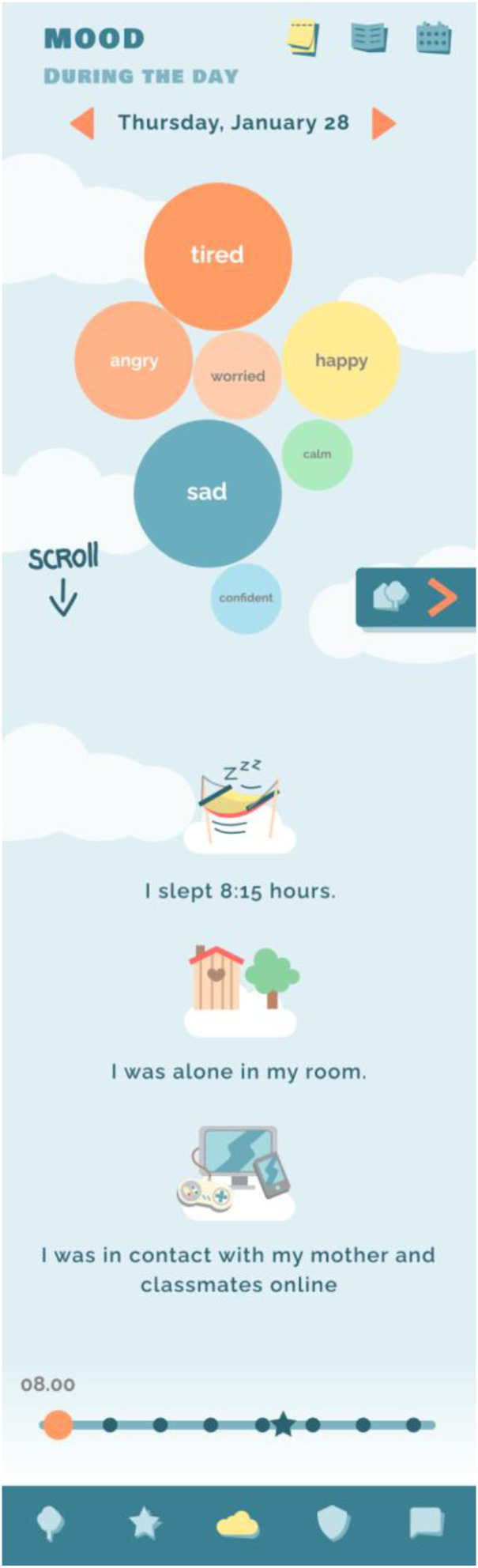
Real-time mood profile Grow It!. *Note: The bigger a sphere, the more present an emotion. There is a possibility to scroll throughout a specific day or week.

**Figure 3.**
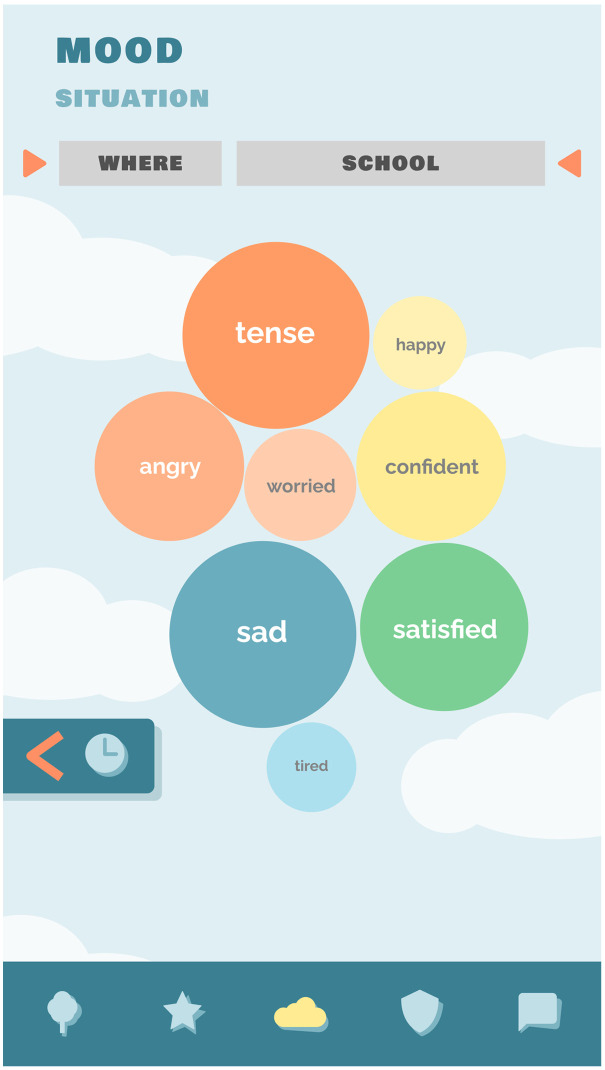
Real-time mood profile Grow It!. *Note: The bigger a sphere, the more present an emotion. There is a possibility to filter real-time on all filled out ESMs across context and situation (e.g. average mood profile when with friends).

The renewed version of Grow It! presents a new feature in the app. As presenting data to teens can be challenging and requires creative ways of presentation which appeal to their level of numerical understanding, we quickly realized that the youth did not understand charts with x-axis or y-axis, typically used in methods such as the life chart. Therefore, we organized a focus group with the aim to co-create an understandable real-time personalized mood profile.^15^ The final visualization of emotional well-being in Grow It! is co-created with psychiatrists that specialized in child and adolescent mental health, developmental and clinical psychologists, data analysts, game designers, and a youth test panel aged 12–25 years based on the circumplex model of affect.^21^ (See Figures 1, 2 and 3). The circumplex model of affect categorizes emotions along two primary dimensions: valence (pleasantness) and arousal (intensity). Emotions are plotted within a circular space, offering a comprehensive framework for understanding emotional experiences.^21^ In Grow It! bigger/darker circles represent a higher mean level of a specific emotion, whereas positioning further away from the center are based on the intensity of the emotions. When children move the ruler through time or when they select a different context, they see dynamical changes in their emotions.

## Measures

### The measures were identical in both samples

#### Affective and cognitive well-being

To measure well-being two questions were asked in the online baseline and follow-up questionnaire: “How happy did you feel last week?” (affective well-being) and “How content with your life were you last week?” [cognitive well-being;^22, 23^]. Affective well-being is scored on a seven-point Likert scale with a score range of 1 to 7 where 1 = not at all, 4 = a little bit, 7 = totally. The item for cognitive well-being was scored on a 10-point Likert scale with a score range of 0 to 10 where 0 = not at all, 5 = a little bit, 10 = totally. Higher scores on affective and cognitive well-being reflect higher affective and cognitive well-being. Previous work shows good convergent validity and good reliability of this single item measurement of affective and cognitive well-being.^6, 23^

### App engagement

App engagement was operationalized as commonly done in behavioral science literature as behavior related to app usage (Perski et al., 2017). In the Grow It! app, this is the percentage of completed ESM questionnaires and the percentage of completed daily challenges.

### User experience

Following guidelines to measure user experience,^24^ evaluation of the app was measured in the follow-up questionnaire by asking participants how they would rate the app and the design of the app (answer scale from 1 to 10) and whether they would recommend the Grow It! app to their friends (yes/no).

## Statistical analysis

All research questions and hypotheses are preregistered at the OSF (labels of hypotheses were slightly adjusted). For RQ1 and RQ2, we assessed differences in app engagement between users of the renewed version of Grow It! versus users who received no personalized feedback in terms of (*H1a*) compliance (*H1b*) challenges and user experience in terms of (*H2a*) user evaluation, (*H2b*) evaluation of design, (*H2c*) recommendability of the app to friends. Independent sample *T*-tests^25^ and a χ^2^-test^25^ were used, accompanied by effect sizes using Cohen's *d* (small *d* = 0.2, medium: *d* = 0.5, large: *d* = 0.8).

For RQ3–RQ5 (only in Sample B), we assessed how the renewed version of the app functioned (following up on earlier publications on older version). Paired samples *T*-tests were conducted to compare cognitive and affective well-being at baseline from cognitive and affective well-being respectively at follow-up (*H3a*). Furthermore, we reported (*H4a*) for how many adolescents a theoretically and clinically relevant change (.20 SD) in well-being had occurred.^26^ Finally, for RQ5, (*H5a*) app engagement and (*H5b*) user experience were related (with the Spearman correlation) to affective and cognitive well-being change rates. We applied α = .05 in all analyses; all *P*-values reported are two-sided. Analyses were performed using the IBM SPSS version 25 for Windows.^27^ The reported results were carefully checked with the stats-check software.^28^

### Sensitivity analyses

As demographic variables across Sample A and Sample B differed significantly (See Supporting information S2) we have added, apart from our preregistered analyses, several sensitivity analyses (regression framework) to our supplemental material (Supporting information S6–S8).

### Missing data and power

Users who did not show any activity in the app (0 ESM and 0 challenges completed) were excluded. Otherwise, all available data were included. For a more in depth overview of attrition, consult Supplementary information Table S1 which includes descriptive statistics for responders and non-responders. Based on the G*power analysis, to achieve power of 0.8 with small, medium, and large effect sizes, a minimum of *N* = 620 was needed; hence, this criterion was met for RQ1 and RQ2 (see Supplementary information Table S2). For RQ3 also the minimum required sample size (*N* = 12, *N* = 27) for a power of 0.8 and large and medium effect sizes, respectively, was met.

### Additional perspective: interviews with health-care professionals

To better understand the perspective of clinicians with regard to the added value of personalized feedback in an mHealth tool and to assess potential barriers seen by professionals for implementation in practice, a convenience sample of nine health-care professionals from different disciplines (i.e. nurses, physiatrists, psychologists, physicians) was invited for interviews between 11 July and 1 August 2022. Nine health-care professionals (9/9; 100% response rate) responded positively to our invite, and after being informed about the purpose of the interviews and the voluntary nature of their participation, they signed informed consent. For detailed information on the sample and data processing see Supplementary Information Table S3 and Appendix S4).

### Ethical approval

The Grow It! study was conducted in accordance with the guidelines proposed in the World Medical Association Declaration of Helsinki and has been approved by the Medical Ethical Committee of the Erasmus Medical Centre (registration number: MEC2020-0287).

## Results

### The added value of personalized feedback

In this real-world unmonitored study of the renewed version of Grow It! (Sample B, *N* = 386), on average 24% (SD = 25.42) of the ESM questions were answered (25 out of 105 ESM notifications), and 41% of the challenges were conducted (nine out of 21 challenges). All participants in Sample B received a real-time emotion profile independently of the number of filled out ESM questions, which were continuously updated with data during the app usage. In general, users were satisfied with the renewed version of the app (scoring 7.22 out of 10 for the app in general, 8.10 for its design, and 66% would recommend it to peers). However, in contrast to our hypotheses, (RQ1 and RQ2) app engagement and user experience were not significantly higher among users who received a personalized real-time mood profile versus those who did not (Table 1). This counts for ESM compliance (*t*(1750,400) = 1.39, *P *= .206, *d* = 0.07), the number of challenges (*t*(692,905) = 0.36, *P *= .971, *d* = 0.00), the overall evaluation of the app (*t*(177,596) = 0.21, *P *= .831, *d* = 0.02), the design of the app (*t*(794) = 1.28, *P *= .202, *d* = 0.12) and recommendability of the app to friends (χ^2^ (659,141) = 2.83, *P *= .091).

**Table 1.** Differences in app engagement and user experience.

	Sample A	Sample B (renewed version)		Test-statistic	*P value*	Effect size (*d*)
**App engagement (RQ1)**	*N* = 1269	*N* = 386				
ESM compliance (%) (*H1a*)	25.65 (26.37)	23.78 (22.03)		*t*(750,400) = −1.39	.206	0.07
Challenges (%) (*H1b*)	40.73 (32.29)	40.80 (29.32)		*t*(692,905) = 0.36	.971	0.00
**User experience (RQ2)**	*N* = 659	*N* = 137–141^a^				
User evaluation (*H2a*)	7.19 (1.31)	7.22 (1.70)		*t*(177,596) = 0.21	.831	0.02
Design (*H2b*)	7.94 (1.33)	8.10 (1.38)		*t*(794) = 1.28	.202	0.12
Recommendability (*H2c*)	73.0% yes (481/659)	65.7% yes (93/141)		χ^2^ = 2.83	.091	-

a Not all participants finished the last questions of the online follow-up questionnaire, therefore the sample size differs.

Apart from our preregistered analyses, we ran a sensitivity analyses in a regression framework to control for possible confounders (sex, age, ethnicity, education, COVID-19 stringency index, see Supplementary information Table S5). Results remain similar, that is, when controlling for demographic characteristics—participants do not differ with regard to app engagement and user experience between Sample A and Sample B.

Zooming in on the personalized mood profile, participants evaluated the personalized mood profile in the follow-up questionnaire on average 3.26 (SD = 1.15) on easiness to understand (five-point scale), indicating that they experienced the mood profile to be “quite clear” to “very clear”. Moreover, we also asked participants what the experienced effect of the personalized mood profile was. A total of 33 of the 138 participants (23.9%) indicated that they learned more about themselves, 9.4% (13/138) felt better because of it, and 38.4% (53/138) reflected more on their feelings. Others indicated self-insights such as: *“I learned from the mood-profile that I've been very tired lately and that I need to change my rhythm a bit to improve it”* (20 year old girl). *“I learned that I feel different in various situations and contexts”* (17 year old boy). *“I learned more about how I feel during the day, and by looking back on that I saw a pattern in my emotions”* (13 year old girl). About 13.1% (18/138) indicated that they shared their personalized mood profile with others which was mostly with friends (61%; 11/18 or with family members (39%; 7/18) .

### Changes in well-being after playing Grow It!

**Figure 4.**
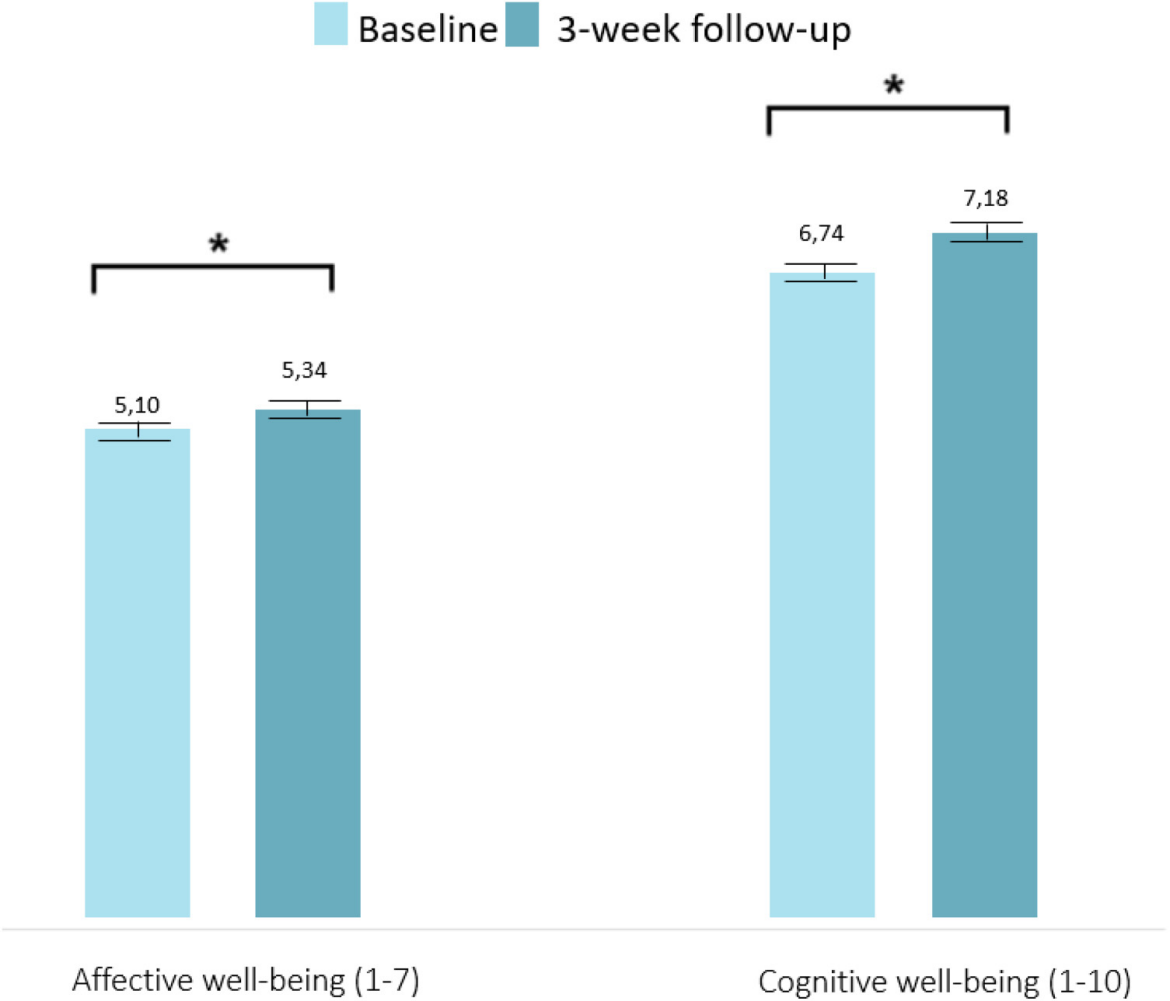
Changes in affective and cognitive well-being from baseline to follow-up in participants who played Grow It! with mood profile.

With regard to the measured effects (RQ3–RQ5), during the 3 weeks of playing Grow It! with personalized feedback (Sample B), adolescents’ affective well-being increased from baseline (mean = 5.07 SD = 1.24) to follow-up (mean = 5.32 SD = 1.27; *t*(175) = 3.01, *P = *.003, *d* = 0.23). Likewise, cognitive well-being increased from baseline (mean = 6.71 SD = 2.20) to follow-up (mean = 7.21 SD = 2.01); *t*(175) = 3.48, *P *= <.001, *d* = 0.26; see also Table 2 and Figure 4). As participants who have a low well-being at baseline could benefit most from Grow It! we reran our analyses with participants below middle on affective and cognitive well-being (see Supplementary material S6). Affective and cognitive well-being still improves significantly in this subgroup of participants. Moreover, 23 participants in our sample recently received treatment for mental health problems we reran our analyses without these participants and found that affective and cognitive well-being still improves in participants (see Supplementary material S7). This result can thus not be explained by the presence of participants that received different treatments at the same time. With regard to clinically meaningful changes (*d* > .2) 38.6–48.9% of the adolescents increased meaningfully in their cognitive and affective well-being. More specifically, 38.6% adolescents increased, 20.5% decreased, and 40.9% remained stable in their affective well-being, and 48.9% adolescents increased, 23.9% decreased, and 27.3% remained stable in their cognitive well-being (i.e. the change was smaller than *d* = 0.2). Neither more positive evaluations (respectively r = .14; r = .02 for affective and cognitive well-being) nor more engagement (compliance: respectively r = .06, r = −.01 for affective and cognitive well-being, and challenges: respectively r = .12, r = .05 for affective and cognitive well-being) correlated significantly with the strength of changes over time in affective and cognitive well-being.

**Table 2.** Changes in well-being before and after Grow It! (Sample B).

Variables	Overall change^a^	*t* test	*P value*	Effect size (*d*)
Affective well-being (RQ3)	+0.25	3.01	.003	0.23
Cognitive well-being (RQ3)	+0.50	3.48	<.001	0.26

a Overall change (mean follow-up–mean baseline).

The effect of Grow It! on affective and cognitive well-being change remained consistent across samples and were not influenced by sex, age, ethnicity, education or COVID-19 sensitivity index, as revealed in our additional sensitivity analyses in Supplementary material S6. Finally, we inspected the influence of baseline well-being scores on change in a regression framework and visualized the individual patterns of change (see Supplementary material S8 and S9).

### The added value of personalized feedback according to health-care professionals

Results of 9 interviews with health-care professionals from different disciplines revealed that all health-care professionals see value in using mHealth tools, such as Grow It!, for their patient populations. Specifically, 9 out of 9 indicated that clients could benefit from the mood profile – and might learn more about their emotional well-being in specific contexts. For example, one health-care professional highlighted that: “…*what I see a lot in young people is that they have limited insight into exactly what they feel, why they feel this, and when they felt this*”. Several benefits from including a mood profile were mentioned by the health-care professionals, including the following codes: *“self-insight”, “monitoring of emotional well-being”, “active involvement in treatment process”, “easy to open a conversation”, “non-stigmatizing”, “better than paper-pencil diaries”, “structured and visual”, “language of the youth”*.

Health-care professionals stress that the mood profile might also have a downside if an adolescent repeatedly reports negative feelings (eight out of nine). “*The feedback is then confronting them with this bad news”.* A solution to this, pointed out by seven out of nine health-care professionals, is to combine mHealth with regular treatment. “*The mood profile can be part of the first, second and later therapy sessions and might lead to new insights for both the patient and myself. If a patient reports negative feelings over the course of several days we can take a look at this pattern together”.* All health-care professionals would like to use the here tested mHealth app in clinical practice for patients specifically with emotional problems.

## Discussion

To promote well-being and mental health among adolescents, innovative new interventions have been developed; several of them capitalizing on the new possibilities offered by mobile technology.^18^ However, when such mHealth interventions are not guided by professionals, adolescents typically have a rather poor engagement and motivation. One possible way to improve this is to provide feedback on their progress and results;^16^ in our case, a real-time mood profile was developed to provide users insights in how they are feeling and doing in their everyday lives. By comparing two versions of our gamified mHealth app, one with and without the added feature of the personalized real-time mood profile, we could test this hypothesis.

The addition of this personal feedback feature was evaluated positively by several users, but comparing different samples of adolescents revealed no improvements in user engagement (i.e. compliance during self-monitoring, executing daily challenges) and user experience (i.e. increased evaluation of the design of the app). Furthermore, we replicated the earlier reported increases in affective and cognitive well-being after playing the app with the mood profile for 3 weeks.^20^ Neither app engagement nor user experience was related to improvements in cognitive or affective well-being, however. Although health professionals appreciated the personalized mood profile and believed it would resonate with youth and enhance self-awareness, they recommended a blended approach to mitigate potential negative effects of self-monitoring. These findings as well as their potential implications for practice are discussed below.

### Principal results and comparisons with prior work

Personalized real-time feedback: an added value? In many domains, providing feedback on one's daily feelings has shown an effective and helpful component of treatment.^29, 30^ For example, in clinical patients with bipolar disorder, a life chart including records of mood and psychological symptoms has shown to help patients, their family members, and clinicians to monitor patients’ mood swings and impact of life events, sleep, and medication more accurately.^31^ However, the extent to which it may also improve an mHealth app for adolescents, is to the best of our knowledge, unanswered.

One of the major drawbacks of monitoring the self is that it often entails activity and engagement of the client. It may be, in fact, a quite tedious task for participants to report several times per day how one is feeling. Both in ESM research settings^32^ and User Experience design of health apps^24^ it has long been discussed how to motivate users to become more engaged with the application. In this project, we took gamification as our approach^32^ and co-designed a personal real-time mood profile which aligns with the numerical skills of adolescents.

The added value of this feature for mHealth for adolescents was evaluated from different perspectives, i.e. by comparing different groups of users and by interviewing both users themselves and professionals. According to users, the mood profile was clear and understandable, and they rated the visual design app with an 8.1 (out of 10). In open ended questions, we learned that even though the profile was intended for the end-user, about 11% of the users of the Grow It! app used their mood profile as a *conversation starter* about their emotional well-being with others, such as their friends and family. A total of 38% indicated that the profile had helped them to reflect on emotions more deeply. Literature indeed suggests that more insight in emotional well-being might result in more adequate help-seeking behavior.^33^ However, our results did not show that that real-time personalized feedback about one's feelings improves users’ motivation to play the app and increase the evaluation of the app as a whole.

It has been suggested before in review studies^3, 16^ that mHealth may help to prevent and improve mental health problems among youngsters. Findings of the current study replicate earlier findings^20^—after playing Grow It! users reported an increase in affective and cognitive well-being. This finding has now been demonstrated in three independent samples. In contrast to our expectations, neither positive evaluations nor more engagement correlated with changes in affective and cognitive well-being after playing the Grow It! app. We had expected that youths who would be more engaged would improve more strongly. However, under the real-world conditions, in which this app was examined, another effect may be present, which cancels this out. With regard to the engagement, one of the explanations that youth provided themselves in exit interviews is that they stopped when they felt the intervention had reached its effects (*“I have learned enough”, “I feel better now”, “I don't need it anymore*”).

Another possible explanation is that the effect of feedback might become more apparent after 6 months, a result that was found by Kramer and colleagues (2014).^11^ Future research, with longer follow-ups, is needed to establish whether the amount of the personal burden may in fact be a motivator for engagement, whether perhaps shorter intervention may already be effective for youth with fewer problems, and how long it takes for effects to become visible.

Based on similar usages of mHealth with self-monitoring, one possible missing factor in our current design is deeper reflection on the meaning of the profiles—which could be achieved in a blended approach. In other programs in which ESM is part of clinical care [e.g.^11–13^] professionals successfully help the client to read and understand the insights from mood profiles. For example in Kramer et al., (2019) depressed out patients have face-to-face sessions in which feedback is provided by a psychologist or psychiatrist on their momentary affective states in specific daily life context and the association with depressive symptoms.^11^

Such potential of the implementation of mHealth as integrated part of treatments was also echoed by the interviewed professionals. According to them, the Grow It! app could have a place in a blended treatment (combination of offline treatment with validated mHealth tools). Potentially, increased self-monitoring could increase self-awareness, which could have a positive effect on the motivation of patients for treatment.^34^ Whereas we did find that most adolescents reported increased well-being—the professionals also highlighted potentially negative effects when the use is unguided. The fact that 19.8–24.0% of the players decreased in well-being could potentially be related to this phenomenon. Hence, an open question which future research needs to address is for whom mHealth can be used safely without access to a health-care provider, and for whom it is best applied in the context of a client–professional relationship. Potentially, rather than showing both positive and negative affect in a mood profile literature suggests that adolescents benefit more from only positive feedback.^35^

### Limitations and future directions

Even though this preregistered study was one of the first evaluations of the added value of personalized real-time feedback module within an innovative gamified mHealth application for youth, some limitations need to be considered. First, data collection of our study took place during the COVID-19 pandemic. This was a period in time in which mHealth was urgently needed in order to prevent mental health problems. However, whether these findings generalize beyond this situation is an open question. Moreover, generalizability might also be influenced by the fact that our sample is skewed toward highly educated female participants, and our study only took place in the Netherlands. However, the study is delivered in real-world every day setting, with little to no exclusion criteria. Therefore our participants might also inform us about the group of youth that are open for this specific intervention (e.g. more girls than boys, higher educated). However, it is also possible that some children or adolescents were less inclined to register due to inadequate digital literacy, yet this is a minor concern as the majority of adolescents in the Netherlands own a mobile device. Furthermore, to equally compare the two samples, matching participants based on characteristics would have been better to provide control for confounding variables. Therefore sensitivity analyses were carried out and revealed similar results (controlled for sex, age, ethnicity, and education). Moreover, we cannot completely rule out that the detected effects are a result of regression to the mean. Even though, this pre–post test design was only one first step toward assessing the app’s effectiveness in real-world settings, an Randomized Controlled Trial is needed to also establish with scientific rigor what the tools’ efficacy is.

Second, compliance was suboptimal and lower than the more fundamental research using ESM in which it is common practice for researchers to regularly contact participants when they miss out assessments and to increase monetary rewards with higher compliance rates.^17, 36^ In exit interviews, we learned that low compliance could have two meanings: it might be a sign of less engagement or in fact a sign of recovery. Third, we did not ask participants whether they perceived their feedback as accurate, which may impact its effectiveness.^35^ The impact of the mood profile may be influenced by the compliance rate, as filling out more ESM questionnaires enhances the accuracy and reliability of the mood profile, potentially leading to increased self-insight.

Finally, the operationalization of app engagement (ESM compliance and completed challenges) is very specific for our app, and it remains unclear whether engagement is of great importance when it comes to the effectivity of an mHealth application.^37^

## Conclusions

Even though mHealth may be a promising innovation for future child and adolescent mental health services, getting youngsters engaged and motivated is a notorious challenge. It has been suggested that real-time feedback about user's own data of how they are feeling may work as a motivational factor, keeping them engaged to return to the app—a hypothesis which did not receive much support here. In our gamified mHealth tool Grow It!, we co-created adolescent feedback reports, which they could access real-time. The effectiveness of the app, in terms of improved well-being, was retained but was still modest. This finding, that personalized feedback may be a useful component of mHealth apps, was substantiated by positive feedback given both by youth and by the health professionals that we interviewed about the potential for clinical practice. However, further improvements are needed in order to meet the full potential of mHealth both for preventing emotional problems and for use within clinical settings.

## Supplemental Material

sj-docx-1-dhj-10.1177_20552076241247937 - Supplemental material for Real-time personalized feedback in mHealth for adolescents Supplemental material, sj-docx-1-dhj-10.1177_20552076241247937 for Real-time personalized feedback in mHealth for adolescents by Evelien Dietvorst, Manon HJ Hillegers, Jeroen S Legerstee, Lianne P De Vries, Annabel Vreeker and Loes Keijsers in DIGITAL HEALTH

## References

[1] Fegert JM Kehoe LA Çuhadaroglu Çetin F , et al. Next generation Europe: a recovery plan for children, adolescents and their families. Eur Child Adolesc Psychiatry 2021; 30: 991–995. 33837857 10.1007/s00787-021-01767-w PMC8035055

[2] Revet A Hebebrand J Anagnostopoulos D , et al. Perceived impact of the COVID-19 pandemic on child and adolescent psychiatric services after 1 year (February/March 2021): ESCAP CovCAP survey. European Child and Adolescence Psychiatry 2021; 32: 249–256. 10.1007/s00787-021-01851-1 PMC8318839 34322720

[3] Clarke AM Kuosmanen T Barry MM . A systematic review of online youth mental health promotion and prevention interventions. Journal of Youth Adolescence 2015; 44: 90–113. 25115460 10.1007/s10964-014-0165-0

[4] Bergin AD Vallejos EP Davies EB , et al. Preventive digital mental health interventions for children and young people: a review of the design and reporting of research. NPJ Digital Med 2020; 3: –9. 10.1038/s41746-020-00339-7 PMC7562906 33083568

[5] Grist R Porter J Stallard P . Mental health mobile apps for preadolescents and adolescents: a systematic review. J Med Internet Res 2017; 19: e176. 10.2196/jmir.7332 PMC5465380 28546138

[6] Beyens I Pouwels JL van Driel II , et al. The effect of social media on well-being differs from adolescent to adolescent. Sci Rep 2020; 10: 1–11. 32612108 10.1038/s41598-020-67727-7 PMC7329840

[7] Franco OH Calkins ME Giorgi S , et al. Evidence for feasibility of mobile health and social media-based interventions for early psychosis and clinical high risk. JMIR Formative Research 2022; 6: e30230. 10.2196/30230 PMC9308069 35802420

[8] Weisel KK Fuhrmann LM Berking M , et al. Standalone smartphone apps for mental health—a systematic review and meta-analysis. NPJ digital Medicine 2019; 2: 1–10. 31815193 10.1038/s41746-019-0188-8 PMC6889400

[9] Dietvorst E Aukes MA Legerstee JS , et al. A smartphone serious game for adolescents (Grow It! app): development, feasibility, and acceptance study. JMIR Formative Research 2022a; 6: 1–16. 10.2196/29832 PMC8931650 35238795

[10] Välimäki M Anttila K Anttila M , et al. Web-based interventions supporting adolescents and young people with depressive symptoms: systematic review and meta-analysis. JMIR Mhealth Uhealth 2017; 5: e180. 10.2196/mhealth.8624 PMC5741826 29222079

[11] Kramer I Simons CJ Hartmann JA , et al. A therapeutic application of the experience sampling method in the treatment of depression: a randomized controlled trial. World Psychiatry 2014; 13: 68–77. 24497255 10.1002/wps.20090 PMC3918026

[12] Bos FM von Klipstein L Emerencia AC , et al. A web-based application for personalized ecological momentary assessment in psychiatric care: user-centered development of the PETRA application. JMIR Ment Health 2022; 9: e36430. 10.2196/36430 PMC9399881 35943762

[13] Nap-van der Vlist MM Houtveen J Dalmeijer GW , et al. Internet and smartphone-based ecological momentary assessment and personalized advice (PROfeel) in adolescents with chronic conditions: a feasibility study. Internet Interv 2021; 25: 100395. 34026566 10.1016/j.invent.2021.100395 PMC8131314

[14] Jeminiwa RN Hohmann NS Fox BI . Developing a theoretical framework for evaluating the quality of mHealth apps for adolescent users: a systematic review. J Pediatr Pharmacol Ther 2019; 24: 254–269. 31337988 10.5863/1551-6776-24.4.254 PMC6633275

[15] Scholten H Granic I . Use of the principles of design thinking to address limitations of digital mental health interventions for youth. J Med Internet Res 2019; 21: e11528. 10.2196/11528 PMC6682276 31344671

[16] Bakker D Kazantzis N Rickwood D , et al. Mental health smartphone apps: review and evidence-based recommendations for future developments. JMIR Ment Health 2016; 3: e7. 10.2196/mental.4984 PMC4795320 26932350

[17] van Roekel E Keijsers L Chung JM . A review of current ambulatory assessment studies in adolescent samples and practical recommendations. Journal of Research on Adolescence 2019; 29: 560–577. 31573762 10.1111/jora.12471 PMC6790669

[18] Smith KE Juarascio A . From ecological momentary assessment (EMA) to ecological momentary intervention (EMI): past and future directions for ambulatory assessment and interventions in eating disorders. Curr Psychiatry Rep 2019; 21: –8. 10.1007/s11920-019-1046-8 31161276

[19] Knaup C Koesters M Schoefer D , et al. Effect of feedback of treatment outcome in specialist mental healthcare: meta-analysis. Br J Psychiatry 2009; 195: 15–22. 19567889 10.1192/bjp.bp.108.053967

[20] Dietvorst E Legerstee JS Vreeker A , et al. The Grow It! app—longitudinal changes in adolescent well-being during the COVID-19 pandemic: a proof-of-concept study. Eur Child Adolesc Psychiatry 2022b; 32: 1097–1107. 35524826 10.1007/s00787-022-01982-z PMC9076805

[21] Russell J . A circumplex model of affect. J Pers Soc Psychol 1980; 39: 1161–1178.

[22] Diener E Oishi S . Handbook of well-being. Amsterdam: Elsevier, 2018.

[23] Cheung F Lucas RE . Assessing the validity of single-item life satisfaction measures: results from three large samples. Qual Life Res 2014; 23: 2809–2818. 24890827 10.1007/s11136-014-0726-4 PMC4221492

[24] Schrepp M . User experience questionnaire handbook. All you need to know to apply the UEQ successfully in your project 2015.

[25] Field A . Discovering statistics using IBM SPSS statistics. sage, 2013. https://www.researchgate.net/profile/Martin-Schrepp/publication/303880829_User_Experience_Questionnaire_Handbook_Version_2/links/575a631b08ae414b8e460625/User-Experience-Questionnaire-Handbook-Version-2.pdf.

[26] Grice JW Medellin E Jones I , et al. Persons as effect sizes. Advances in Methods and Practices in Psychological Science 2020; 3: 443–455.

[27] *IBM Corp* . IBM SPSS Statistics for Windows, Version 25.0. Armonk, NY: IBM Corp, Released 2017.

[28] Rife SC Nuijten MB Epskamp S . Statcheck: Extract statistics from articles and recompute p-values [web application]. Retrieved from http://statcheck.io. 2016.

[29] Cohen JS Edmunds JM Brodman DM , et al. Using self-monitoring: implementation of collaborative empiricism in cognitive-behavioral therapy. Cogn Behav Pract 2013; 20: 419–428.

[30] Tarrier N Sommerfield C Reynolds M , et al. Symptom self-monitoring in the treatment of posttraumatic stress disorder. Behav Ther 1999; 30: 597–605.

[31] Denicoff KD Leverich GS Nolen WA , et al. Validation of the prospective NIMH-life-chart method (NIMH-LCMTM-p) for longitudinal assessment of bipolar illness. Psychol Med 2000; 30: 1391–1397. 11097079 10.1017/s0033291799002810

[32] Van Berkel N Goncalves J Hosio S , et al. Gamification of mobile experience sampling improves data quality and quantity. Proceedings of the ACM on Interactive, Mobile, Wearable and Ubiquitous Technologies 2017; 1: 1–21.

[33] Collin PJ Metcalf AT Stephens-Reicher JC , et al. Reachout. Com: the role of an online service for promoting help-seeking in young people. Advances in Mental Health 2011; 10: 39–51.

[34] Habets J Heijmans M Herff C , et al. Mobile health daily life monitoring for Parkinson disease: development and validation of ecological momentary assessments. JMIR Mhealth Uhealth 2020; 8: e15628. 10.2196/15628 PMC7248801 32339999

[35] Leertouwer I Cramer AO Vermunt JK , et al. A review of explicit and implicit assumptions when providing personalized feedback based on self-report EMA data. Front Psychol 2021; 12: 39–51. 10.3389/fpsyg.2021.764526 PMC8693716 34955984

[36] Myin-Germeys I Kuppens P . The open handbook of Experience Sampling Methodology (2nd ed.). The center for Research on Experience sampling and Ambulatory methods Leuven (REAL) - Belgium. 2022.

[37] Nahum-Shani I Shaw SD Carpenter SM , et al. Engagement in digital interventions. Lausanne, Switserland: American Psychologist, 2022. 10.1037/amp0000983 PMC9481750 35298199

