# Supplementary material for: Real-time personalized feedback in mHealth for adolescents
Source: Digit Health. 2024 May 15;10:20552076241247937. doi: 10.1177/20552076241247937 (PMC11100393; doi:10.1177/20552076241247937)
Supplement: sj-docx-1-dhj-10.1177_20552076241247937 - Supplemental material for Real-time personalized feedback in mHealth for adolescents [file sj-docx-1-dhj-10.1177_20552076241247937.docx]

# Supporting Information

**Table S1. Descriptive variables of responders and non-responders**

**Table S2. Differences in demographic characteristics among Sample A and Sample B**

**Table S3. A priori power calculation**

**Table S4. Sample characteristics of health-care professionals**

**Appendix S5. Interview Guide**

**Table S6 Sensitivity analyses**

**Table S7. Changes in well-being before and after Grow it! (Sample B) of participants who scored below middle on affective or cognitive well-being**

**Table S8. Sensitivity analyses: Changes in well-being before and after Grow it! (Sample B)**

**Figure S9. Change Score Visualizations: Exploring Well-being Outcomes in Samples A-B with Group Segmentation**

**Table S10. Prediciting change in well-being with baseline well-being**

**Table S1. Descriptive statistics responders and non-responders**

|  | **Sample A** | | | **Sample B** | | |
| --- | --- | --- | --- | --- | --- | --- |
|  | N=1269 | Non-responders  (N=610) | Responders follow-up (N= 659, 52%) | N=386 | Non-responders (N=245) | Responders follow-up (N= 137-141, 36-37%) |
| **Age (years), mean (SD)** | 18.60 (3.39) | 18.93 (3.51) | 18.33 (3.28) | 16.04 (3.21) | 16.15 (3.33) | 15.91 (3.07) |
| **Sex, (%girls)** | 80.6% | 78.3% | 81.1% | 67.6% | 71.0% | 64.8% |
| **Education^a^ level, (%)** |  |  |  |  |  |  |
| Low | 17.9% | 20.0% | 16.5% | 8.1% | 5.7% | 10.1% |
| Medium | 35.1% | 37.8% | 32.5% | 19.9% | 17.2% | 22.1% |
| High | 46.6% | 40.5% | 50.6% | 70.2% | 74.1% | 66.8% |
| Other | 0.9% | 1.8% | 0.5% | 1.8% | 2.9% | 1.0% |
| **Cultural Identity** |  |  |  |  |  |  |
| Dutch | 95.4% | 93.1% | 96.3% | 82.9% | 82.4% | 83.3% |
| **Baseline Affective wellbeing** | 4.22 (1.38) | 4.16 (1.42) | 4.27 (1.34) | 4.94 (1.33) | 4.84 (1.40) | 5.07 (1.24) |
| **Baseline Cognitive wellbeing** | 5.42 (2.16) | 5.31 (2.25) | 5.51 (2.08) | 6.62 (2.21) | 6.54 (2.23) | 6.71 (2.20) |

aLow: (preparatory school for) technical and vocational training; medium: (preparatory school for) professional education; and high: (preparatory school for) university.

**Table S2. Differences in demographic characteristics among Sample A and Sample B**

|  | **Sample A (N=1269)** | **Sample B (N=386)** | **Sample A vs Sample B** |
| --- | --- | --- | --- |
| **Age (years), mean (SD)** | 18.60 (3.39) | 16.04 (3.21) | t(1653) = 13.15, p < .001 |
| **Sex, (%girls)** | 80.6% girls | 67.6% | X2(1, N = 1655) = 28.75, p < .001 |
| **Cultural identity** | 95.4% Dutch | 82.9% Dutch | X2(1, N = 1655) = 65.69, p < .001 |
| **Education^a^ level, (%)** | Low 17.9%, 35.1% medium, 46.6% high, 0.9% other | 8.1% low, 19.9% medium, 70.2% high, 1.8% other | X2(1, N = 1655) = 7724, p < .001 |
| **COVID-19 stringency index** | 78.99 (1.39) | 72.22 (3.41) | t(1653) = 56.90, p < .001 |

**Table S3. A priori power calculation**

|  | **Large effect size (0.8)** | **Medium effect size (0.5)** | **Small effect size (0.2)** |
| --- | --- | --- | --- |
| **RQ1 - Difference between two independent means (two groups)** | 21 + 21 =  N=42 | 51 + 51 =  N=102 | 310 + 310 =  N=620 |
| **RQ2- Difference between two independent means (two groups)** | 21 + 21 =  N=42 | 51 + 51 =  N=102 | 310 + 310 =  N=620 |
| **RQ 3 - paired sample T-test** | N=12 | N=27 | N=156 |
| **RQ 4 - individual measures** | taps into individual measures and is descriptive a power analyses is therefore not applicable | | |
| **RQ 5 - Correlational** | In order to detect a correlation of 0.3 a minimal required sample size of N= 67 is required. | | |

**Table S4. Sample characteristics of health-care professionals**

| **Participant number** | **Gender (M/F)** | **Profession** | **Familiar with Grow It! app in scientific context?** |
| --- | --- | --- | --- |
|  | F | PhD in psychology | Yes |
|  | F | Social worker | No |
|  | M | Nurse practitioner mental health care youth | No |
|  | F | Psychiatric nurse | No |
|  | F | Nurse practitioner | Yes |
|  | F | Resident dermatology | Yes |
|  | F | PhD in psychology | No |
|  | M | Resident psychiatry | No |
|  | F | Nurse practitioner | Yes |

***Note. F=female, M=male.***  Interviews were audio taped and conducted by ED (PhD student) and IB (PhD student) and lasted on average 27 minutes (range 22:22 – 37:40 ). The semi-structured interview allowed for flexibility and deeper examination of arising issues, but was mostly carried out according to the interview guide (Supplementary Information Appendix S3). The audio of interviews was transcribed verbatim with participant’s permission. The coder (ED) read all transcripts to familiarize herself with the data. Thereafter, the data was structured in themes and subthemes by the coders using an inductive process [patterns, themes and categories arise from the data; 29]. To ensure confidentiality, all identifying information was removed from the quotes.

**Appendix S5. Interview Guide**

**Semi-structured Interview Schedule: Grow It! Sophia Healthcare Professionals**

Central Research question for interview: What do healthcare professionals think of the mood profile in the Grow It! app and how could it contribute to the recovery of young people?

1. Introduction
   1. Thank you for your participation. Introduction of ED and IB.
   2. Ask participants to explain his or her discipline, expertise and function in health care.
   3. Report age and gender of participants.
   4. Ask participants about the familiarity of Grow It! app. In both cases. Explain the basic game mechanics of the app, including the renewed version.

*Grow It! is a serious gaming app specially developed for adolescents at risk for emotional problems. An example of such a high risk adolescent can be an adolescent that suffers from a chronic disease, or an adolescent who has a parent with psychiatric problems.*

*Grow It! consist of (1) daily diary method in which participants are asked to report on their emotional wellbeing five times per day (2) daily challenges such as “ask your mom what she likes about you and write it down”, with the aim of stimulating active coping.*

*Recently, we have added a new feature to our app: personal feedback in the form of a mood profile (show image). In this profile, participants get an overview of their emotional wellbeing after they fill out the daily diaries. In real-time this profile is filled with the data that participants provide. The more an emotion is present, the larger the icons are. In this case a participant is for example mostly tired and sad, and not so happy. Within the mood profile it is possible to filter on context, specific days and weeks. At the end of the Grow It! app study, participants also received an individualized mood profile report. This contains all daily dairy data – in the form of graphs – in a PDF that can easily be shared. Participants are the owners of their own data.*

*So far, we have only used Grow It! in the context of scientific research. We would like to talk with you about Grow It! and the possibility of implementing Grow It! in clinical care (for the youth you work with).*

| **Topics** | **Question** |
| --- | --- |
| Personal feedback in the form of a mood profile | What do you think about the mood profile in relation to the population of patients that you treat? |
| Patient Perspective | What do you think would be the advantages and disadvantages of personalized feedback for your patients? |
| Clinician's Perspective | What do you think would be the advantages and disadvantages of personal feedback for you as a healthcare professional? |
| Application, recovery | How would you like to apply the personal feedback in your area of ​​expertise? |

**Table S6. Sensitivity analyses**

| **Model** | **Predictor** | **Beta** | ***p*** | **Adjusted R^2^** | ***p*** |
| --- | --- | --- | --- | --- | --- |
| **[DV ESM Compliance]** | | | | | |
| 1 |  |  |  | 0.019 | <.001* |
|  | Sex | -.103 | <.001* | - | - |
|  | Ethnicity | -.007 | .753 | - | - |
|  | Education | .094 | <.001* | - | - |
|  | Age | -.003 | .904 |  |  |
|  | COVID-19 stringency | .028 | .234 | - | - |
| 2 |  |  |  | 0.019 | <.001* |
|  | Sex | -.104 | <.001* |  |  |
|  | Ethnicity | -.005 | .830 | - | - |
|  | Education | .095 | <.001* | - | - |
|  | Age | -.006 | .811 |  |  |
|  | COVID-19 stringency | .000 | .999 |  |  |
|  | Sample A vs B | -.036 | .366 |  |  |
| **[DV Challenges]** | | | | | |
| 1 |  |  |  | 0.024 | <.001* |
|  | Sex | -.118 | <.001* | - | - |
|  | Ethnicity | .005 | .834 | - | - |
|  | Education | .093 | <.001* | - | - |
|  | Age | -.084 | <.001* |  |  |
|  | COVID-19 stringency | .030 | .208 | - | - |
| 2 |  |  |  | 0.024 | <.001* |
|  | Sex | -.119 | <.001* |  |  |
|  | Ethnicity | .007 | .777 | - | - |
|  | Education | .094 | <.001* | - | - |
|  | Age | -.086 | <.001* |  |  |
|  | COVID-19 stringency | .008 | .832 |  |  |
|  | Sample A vs B | -.028 | .490 |  |  |
| **[DV user experience]** | | | | | |
| 1 |  |  |  | .045 | <.001* |
|  | Sex | .004 | .908 | - | - |
|  | Ethnicity | -.001 | .983 | - | - |
|  | Education | -.107 | .003 | - | - |
|  | Age | -.201 | <.001* |  |  |
|  | COVID-19 stringency | -.005 | .885 | - | - |
| 2 |  |  |  | .046 | <.001* |
|  | Sex | .004 | .920 |  |  |
|  | Ethnicity | .006 | .859 | - | - |
|  | Education | -.102 | .005 | - | - |
|  | Age | -.210 | <.001* |  |  |
|  | COVID-19 stringency | -.075 | .213 |  |  |
|  | Sample A vs B | -.091 | .142 |  |  |
| **[DV Design]** | | | | | |
| 1 |  |  |  | 0.049 | <.001* |
|  | Sex | -.114 | .002* | - | - |
|  | Ethnicity | -.019 | .590 | - | - |
|  | Education | .007 | .843 | - | - |
|  | Age | -.222 | <.001* |  |  |
|  | COVID-19 stringency | -.016 | .662 | - | - |
| 2 |  |  |  | 0.049 | <.001* |
|  | Sex | -.115 | .002* |  |  |
|  | Ethnicity | -.015 | .679 | - | - |
|  | Education | .010 | .784 |  |  |
|  | Age | -.227 | <.001* | - | - |
|  | COVID-19 stringency | -.057 | .344 |  |  |
|  | Sample A vs B | -.053 | .389 |  |  |
| **[DV affective well-being change]** | | | | | |
| 1 |  |  |  | 0.001 | .346 |
|  | Sex | -.040 | .256 |  |  |
|  | Ethnicity | -.061 | .085 |  |  |
|  | Education | .000 | .995 |  |  |
|  | Age | -.007 | .842 |  |  |
|  | COVID-19 stringency | .027 | .469 |  |  |
| 2 |  |  |  | 0.000 | .461 |
|  | Sex | -.040 | .258 |  |  |
|  | Ethnicity | -.062 | .082 |  |  |
|  | Education | -.001 | .987 |  |  |
|  | Age | -.006 | .875 |  |  |
|  | COVID-19 stringency | .038 | .518 |  |  |
|  | Sample A vs B | .015 | .800 |  |  |
| **[DV cognitive well-being change]** | | | | | |
| 1 |  |  |  | -.006 | .997 |
|  | Sex | .007 | .836 |  |  |
|  | Ethnicity | -.014 | .701 |  |  |
|  | Education | -.007 | .843 |  |  |
|  | Age | -.001 | .969 |  |  |
|  | COVID-19 stringency | .007 | .840 |  |  |
| 2 |  |  |  | -.006 | .966 |
|  | Sex | .008 | .827 |  |  |
|  | Ethnicity | -.018 | .611 |  |  |
|  | Education | -.010 | .773 |  |  |
|  | Age | .004 | .904 |  |  |
|  | COVID-19 stringency | .056 | .347 |  |  |
|  | Sample A vs B | .063 | .299 |  |  |

**Note.** Sensitivity analyses was run in a regression framework. For all outcomes (ESM compliance, challenges, user evaluation, design, change score affective well-being, change score cognitive well-being) we added the possible confounders to the regression (sex, age, ethnicity, education, COVID-19 stringency index). In a second step we added the variable that indicated to which sample a participant belonged (Sample A vs sample B) to the model.

In summary, participants who played the Grow It! app with the mood profile did not have higher app engagement nor user experience. When controlling for demographic characteristics (which differ among Sample A and Sample B) we find similar results: Participant who played the Grow It! app with the mood profile did not have higher app engagement nor user experience. Moreover, we have found no evidence indicating that the differences in demographic characteristics influenced the difference scores affective and cognitive well-being.

**Table S7. Changes in well-being before and after Grow it! (Sample B) of participants who scored below middle on affective or cognitive well-being**

| **Variables** | **Overall change^a^** | ***t* test** | ***P value*** | **df** |
| --- | --- | --- | --- | --- |
| **Affective well-being (RQ3)** | **+0.80** | **3.75** | **<.001** | **43** |
| **Cognitive well-being (RQ3)** | **+1.72** | **5.52** | **<.001** | **49** |

**aOverall change (mean follow-up – mean baseline)**

**Table S8. Sensitivity analyses: Changes in well-being before and after Grow it! (Sample B)**

|  | **Overall change^a^** | ***t*-test** | ***P*** | **Effect size (*d*)** |
| --- | --- | --- | --- | --- |
| Affective well-being (RQ3) | +26 | 3.10 | .002 | .24 |
| Cognitive well-being (RQ3) | +56 | 3.89 | <.001 | .30 |

^a^Overall change (mean follow-up – mean baseline). *Note: analysis performed without participants that recently received treatment for mental health problems.*

**S9. Change Score Visualizations: Exploring Well-being Outcomes in Samples A-B with Group Segmentation**


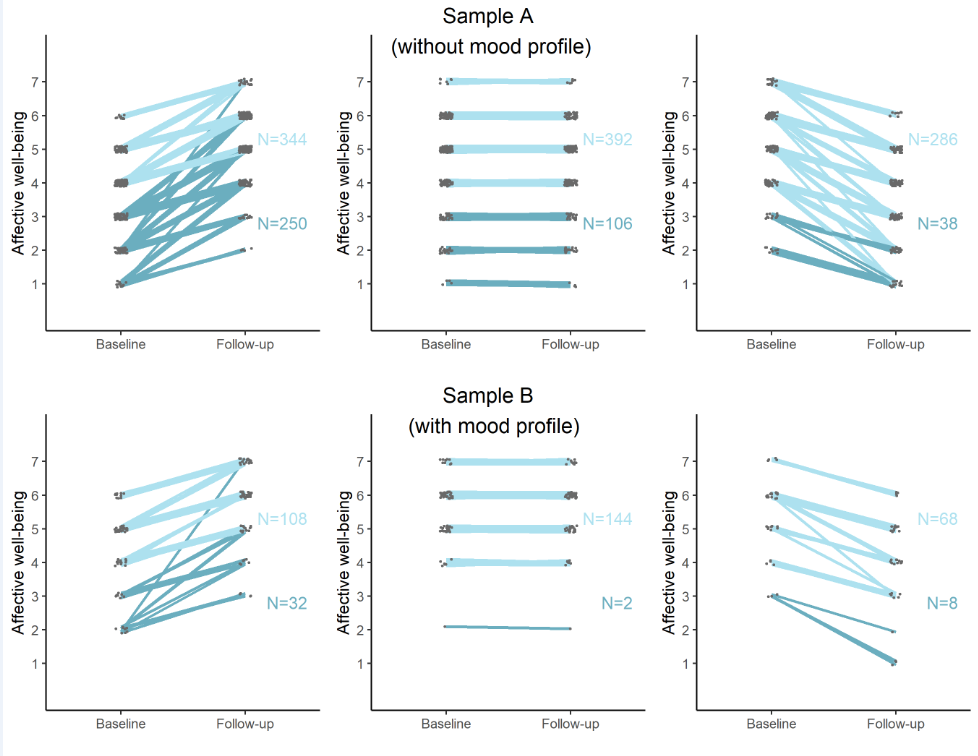


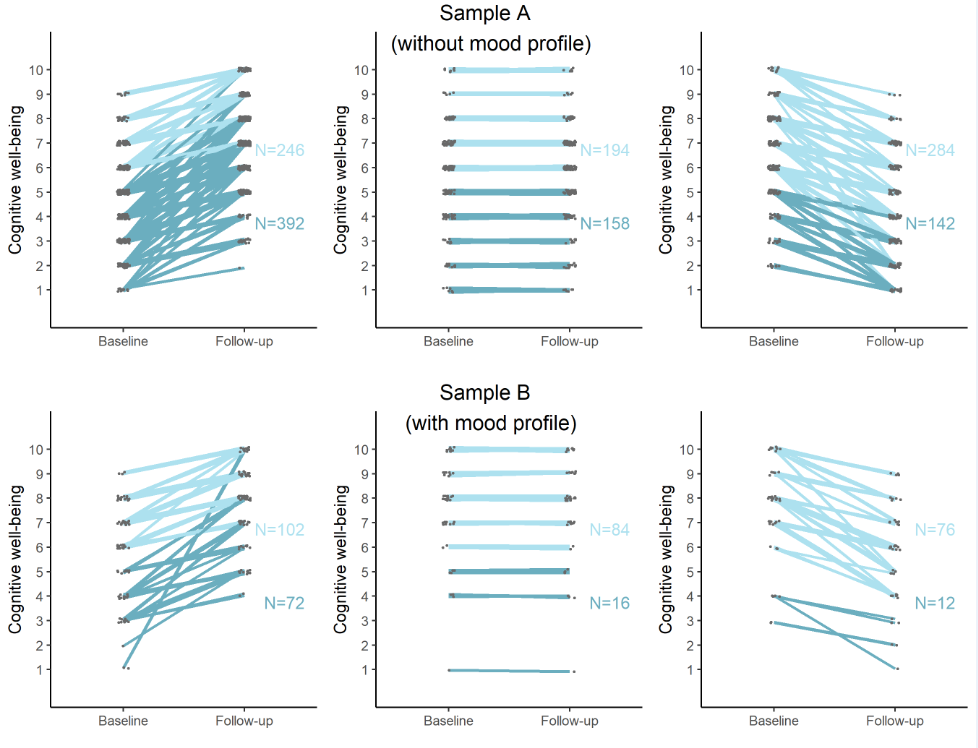


**S10. Prediciting change in well-being with baseline well-being**

| **Model** | **Predictor** | **Beta** | **p** | **Adjusted R2** | **p** |
| --- | --- | --- | --- | --- | --- |
| Sample B [DV Change in affective well-being] | | | | | |
| **1** |  |  |  | .17 | <.001* |
|  | Baseline affective well-being | -.42 | <.001* | - | - |
| Sample B [DV Change in cognitive well-being] | | | | | |
| **1** |  |  |  | .28 | <.001* |
|  | Baseline cognitive well-being | -.53 | <.001* | - | - |
